# A SIGNAL FOR HEALTH EQUITY: DIFFERENCES IN WHOLE HEALTH AND SOCIAL NEEDS AMONG MEDICARE ADVANTAGE SUBPOPULATIONS

**DOI:** 10.1093/geroni/igad104.3153

**Published:** 2023-12-21

**Authors:** Kelsey McNamara, Ellen Rudy

**Affiliations:** Papa Inc., Miami, Florida, United States; Papa Inc., Miami, Florida, United States

## Abstract

CMS’ 2024 Medicare Advantage (MA) Final Rule introduced the health equity index (HEI) to encourage plans to advance equity and improve outcomes for enrollees with social risk factors. Enrollees of focus include those receiving a low-income subsidy, dually eligible for Medicare and Medicaid, and/or having a disability. These populations historically have higher unmet social needs and poorer health outcomes. The purpose of this epidemiological study was to compare self-reported whole health and social needs among MA subpopulations. Analysis included 28,588 MA members who were screened for a companion care program (January–June 2023). Questionnaire included a social needs screener, UCLA-3 Loneliness Scale, and CDC’s Healthy Days Measure. To compare across metrics/domains (mental health, physical health, whole health, social support, loneliness), data was normalized 0 to 100 where higher scores represent better outcomes. Among the cohort, average age was 72, 31% male, and 37 states represented. Subpopulations included 70% traditional MA (TMA), 13% dual-eligible (DE), 17% people with disabilities (PWD), < 1% veterans (V). Average outcomes reported by subpopulations were (TMA; DE; PWD; V): number of unmet social needs (1.6; 2.3; 2.6; 1.4); social support score (63; 54; 53; 70); mental health score (74; 63; 59; 73); physical health score (57; 50; 48; 52); whole health score (65; 56; 53; 65). People with disabilities reported the lowest scores and indicate an important subpopulation in need. Meeting the HEI, and ultimately improving outcomes for some of the most historically marginalized members, will require an individualized and holistic approach to health and social care.

